# Barriers and facilitators for the sustainability of digital health interventions in low and middle-income countries: A systematic review

**DOI:** 10.3389/fdgth.2022.1014375

**Published:** 2022-11-28

**Authors:** Soutongnoma Safiata Kaboré, Patrice Ngangue, Dieudonné Soubeiga, Abibata Barro, Arzouma Hermann Pilabré, Nestor Bationo, Yacouba Pafadnam, Koiné Maxime Drabo, Hervé Hien, Gueswendé Blaise Léon Savadogo

**Affiliations:** ^1^ Centre D'excellence Africain, Université Nazi BONI, Bobo Dioulasso, Burkina Faso; ^2^ Direction Générale, Institut National de Santé Publique, Ouagadougou, Burkina Faso; ^3^ Département des Sciences Infirmières et Obstétricales, Institut de Formation et de Recherche Interdisciplinaires en Sciences de la Santé et de L'Éducation, Ouagadougou, Burkina Faso; ^4^ Département des Sciences de la Santé, Université du Québec en Abitibi Témiscamingue, Québec, Canada; ^5^ Département de Santé Publique, Université Joseph Ki-ZERBO, Ouagadougou, Burkina Faso; ^6^ Institut de Recherche en Sciences de la Santé/Centre National Pour la Recherche Scientifique et Technologique, Ouagadougou, Burkina Faso

**Keywords:** health systems, eHealth, mHealth, sustainability, barriers, facilitators, low and middle-income countries, systematic review

## Abstract

**Background:**

Digital health interventions (DHIs) have increased exponentially all over the world. Furthermore, the interest in the sustainability of digital health interventions is growing significantly. However, a systematic synthesis of digital health intervention sustainability challenges is lacking. This systematic review aimed to identify the barriers and facilitators for the sustainability of digital health intervention in low and middle-income countries.

**Methods:**

Three electronic databases (PubMed, Embase and Web of Science) were searched. Two independent reviewers selected eligible publications based on inclusion and exclusion criteria. Data were extracted and quality assessed by four team members. Qualitative, quantitative or mixed studies conducted in low and middle-income countries and published from January 2000 to May 2022 were included.

**Results:**

The sustainability of digital health interventions is very complex and multidimensional. Successful sustainability of digital health interventions depends on interdependent complex factors that influence the implementation and scale-up level in the short, middle and long term. Barriers identified among others are associated with infrastructure, equipment, internet, electricity and the DHIs. As for the facilitators, they are more focused on the strong commitment and involvement of relevant stakeholders: Government, institutional, sectoral, stakeholders' support, collaborative networks with implementing partners, improved satisfaction, convenience, privacy, confidentiality and trust in clients, experience and confidence in using the system, motivation and competence of staff. All stakeholders play an essential role in the process of sustainability. Digital technology can have long term impacts on health workers, patients, and the health system, by improving data management for decision-making, the standard of healthcare service delivery and boosting attendance at health facilities and using services. Therefore, management changes with effective monitoring and evaluation before, during, and after DHIs are essential.

**Conclusion:**

The sustainability of digital health interventions is crucial to maintain good quality healthcare, especially in low and middle-income countries. Considering potential barriers and facilitators for the sustainability of digital health interventions should inform all stakeholders, from their planning until their scaling up. Besides, it would be appropriate at the health facilities level to consolidate facilitators and efficiently manage barriers with the participation of all stakeholders.

## 1. Background

Today, the term digital health is often used as an umbrella term that takes into account many eHealth areas (1). It encompasses some areas such as advanced computing sciences (in the fields of big data, genomics and artificial intelligence), care and health service providers, patients, public health authorities, universities and research institutions (1). Digital health means “the field of knowledge and practice associated with the development and use of digital technologies to improve health” (1). This definition includes eHealth. Digital health expands the concept of eHealth to include digital consumers, with a wider range of smart and connected devices. It also encompasses other usages of digital technologies for health such as the Internet of Things, advanced computing, big data analytics, artificial intelligence including machine learning, and robotics (1). Technologies such as the Internet of things, virtual care, remote monitoring, artificial intelligence, big data analytics, blockchain, smart wearables, platforms, tools that enable data exchange and storage, tools that enable remote data capture and the exchange of data sharing of relevant information across the health ecosystem creating a continuum of care have proven potential to enhance health outcomes by improving medical diagnosis, data-based treatment decisions, digital therapeutics, clinical trials, self-management of care and person-centred care as well as creating more evidence-based knowledge, skills and competence for professionals to support health care (1).

**Figure 1:**
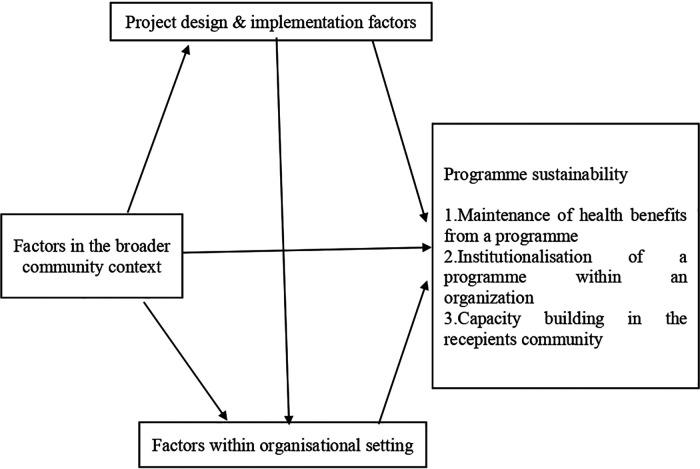
Shediac-Rizkallah and bone framework conceptualising programme sustainability.

These tools enable remote data capture and the exchange of data sharing of relevant information across the health ecosystem (1). DHIs create a continuum of care have proven potential to enhance health outcomes by improving medical diagnosis, data-based treatment decisions, digital therapeutics, clinical trials, self-management of care and person-centred care as well as creating more evidence-based knowledge, skills and competence for professionals to support health care (1). Digital health interventions (DHIs) are defined as information and communication technology to improve health systems and services. This definition deliberately includes concepts of both mobile health (mHealth) and electronic health (eHealth) (2). In the vision of global strategy, it is crucial to strengthen health systems through the application of digital health technologies (DHIs) for consumers, health professionals, health care providers, and industry towards empowering patients and achieving the vision of health for all (1). DHIs play a central role in improving access, quality and efficiency to health care and services (3). Digital technologies shape the future of global health (1). The global adoption of digital technology, including by the poorest people in low and middle-income countries (LMICs), presents a genuine opportunity to reduce inequality in the domain of healthcare system (4). DHIs have proved to be an outstanding solution to addressing the above gaps in health care delivery (5). In LMICs, the number and scope of DHIs are rapidly surged. Despite this rapid improvement, few digital solutions reach national or semi-national scales, and even fewer are used sustainably (6). Despite its importance, few studies have analysed the sustainability of DHIs (7). *Sustainability* of DHIs impacts is defined as the longevity and the continuous manifestation for the benefits and outcomes of digital innovations for health workers, the standard of healthcare, and patient experience long after the end of the program (5). The sustainability of DHIs is unavoidable in LMICs given the double burden of diseases, health care worker shortage, weak health systems, and limited resources (8). Sustainability is a core component of the overall life cycle of interventions implemented (8). Because sustainability cannot be dissociated from the successful implementation and deployment of projects (3). Sustainability is a dynamic process, and that goals and strategies for achieving it must continuously adapt to changing environmental conditions (9). Throughout the world, considerable resources are implementing community-based health programs that are discontinued soon after initial funding ends (10). In recent years, program sustainability has been an issue of growing concern (9). Attention to the long-term viability of health intervention programs is likely to increase everywhere, as policymakers and funders become increasingly concerned by allocating scarce resources effectively and efficiently (9). There are many challenges to the broader implementation and sustainability of DHIs, including the absence of monetary resources, limited visibility outside the healthcare sector, the lack of integration with community-based and nationwide programmes, services and information and communication technologies, and the limited local capacity about the maintenance, further development, and management (7). Faced with this, researchers have taken an interest in maintaining the sustainability of DHIs. A literature review shows several pathways for the sustainability of DHIs have been discussed. Despite the growth in the implementation research, limited scientific attention has focused on understanding and improving the sustainability of health interventions (11). However, a systematic synthesis of DHIs sustainability challenges is lacking, especially in LMICs. Planning for sustainability requires, first, a clear understanding of the concept of sustainability and operational indicators that may be used in monitoring sustainability over time (9). The main objective of this study is to identify the barriers and facilitators for the sustainability of DHIs in LMICs. The scope of this research is relevant because research on the sustainability of health projects in LMICs is scarce as most projects investigate the initial adoption and implementation of an intervention (7). The broader scale-up of interventions is rarely investigated, largely due to constrained timeframes and/or funding for research (11). Recently, there has been interest in understanding and influencing the sustainability of implemented interventions (11). Good sustainability of DHIs can ensure long-term effects and impacts on populations. This research could help all stakeholders to improve the more comprehensive implementation and sustainability of DHIs in LMICs. The potential influences on sustainability may derive from three major groups of factors according to Shediac-Rizkallah and Bone's framework for conceptualising programme sustainability presented in Figure 1: (1) Project design and implementation factors; (2) Factors within the organisational setting, and (3) Factors in the broader community environment (9). In addition to context factors, several project characteristics were related to sustainability (9). Thus, we asked the main following research question: What are the barriers and facilitators for the sustainability of DHIs in LMICs? Secondary questions are: What are the barriers that negatively influence the sustainability of DHIs? What are the facilitators that positively influence the sustainability of DHIs?

## 2. Methods

This systematic review examines the barriers and facilitators for the sustainability of DHIs in LMICs. It was conducted following the Preferred Reporting Items for Systematic reviews and Meta-Analyses PRISMA (12–14).

### 2.1 Eligibility criteria

All original research articles on barriers and facilitators for the sustainability of DHIs meeting the following eligibility criteria were included: (1) Research with a quantitative, qualitative or mixed design; (2) Articles published in English or French; (3) Articles published from January 2000 to May 2022; (4) Limited to low- and middle-income countries. Exclusion criteria were: (1) Commentaries, review papers, case studies, letters, discussion papers, posters, conference abstracts, conference reports, and dissertations; (2) Full text available.

### 2.2 Information sources

A complete search strategy was developed to identify studies published in English or French from January 2000 to May 2022. Three electronic databases were consulted (PubMed, Embase and Web of Science). The search was limited to human subjects.

### 2.3 Search strategies

The search strategy for the Pudmed database was as follows: (“health personnel” [MeSH] OR “nurses” [MeSH] OR “physicians” [MeSH] OR “pharmacists” [MeSH] OR “caregivers” [MeSH] OR “delivery of health care, integrated” [MeSH] OR “health policy” [MeSH] OR “Women” [MeSH] OR “Female” [MeSH] OR “Young Adult” [MeSH] OR “Women of Childbearing Age” OR “Women of Reproductive Age”) AND (“medical informatics” [MeSH] OR “electronic health records” [MeSH] OR “medical order entry systems” [MeSH] OR “computers” [MeSH] OR “technology” [MeSH] OR “Cell Phone*” [MeSH] OR “Health Communication*” [MeSH] OR “Mobile Applications” [MeSH] OR “Text Messaging*” [MeSH] OR “Telephone” [MeSH] OR “Text Messaging” [MeSH] OR “Communications Media” [MeSH] OR “Smartphone” [MeSH] OR “Telecommunications” [MeSH] OR “Telemedicine*” [MeSH] OR “Reminder Systems*” [MeSH] OR “Wireless Technology” [MeSH] OR “medical informatics applications” [MeSH] OR “telemedicine” [MeSH] OR “mobile applications” [MeSH] OR “biomedical technology” [MeSH] OR “digital technology” [MeSH] OR “computer systems” [MeSH] OR “Computerized technological resources” OR “computer communication networks” [MeSH]) AND (“communication” [MeSH] “decision support systems, clinical” [MeSH] OR “Decision aid” OR “Behavior change communication” OR “financial support” [MeSH] OR “policy” [MeSH] “policy making” [MeSH] OR “health policy” [MeSH] OR “motivation” [MeSH] OR “personal satisfaction” [MeSH] OR “patient satisfaction” [MeSH] OR “delivery of health care” [MeSH] OR “adoption” [MeSH] OR “gender identity” [MeSH] OR “self-management” [MeSH] OR “empowerment” [MeSH] OR “education” [MeSH] OR “computer literacy” [MeSH] OR “community participation” [MeSH] “stakeholder participation” [MeSH] OR “patient participation” [MeSH] OR “health information interoperability” [MeSH] OR “social participation” [MeSH] OR “knowledge” [MeSH] “attitude” [MeSH ] OR “stakeholder participation” [MeSH] OR “universal design” [MeSH] OR “data management” [MeSH] OR “built environment” [MeSH] OR “computer security” [MeSH] OR “electricity” [MeSH] OR “internet” [MeSH] OR “professional competence” [MeSH] OR “sustainability”) AND (“Africa South of the Sahara” [MeSH] OR “Sub-Saharan Africa” OR “Africa, Central” [MeSH] OR “Africa, Eastern” [MeSH] OR “Africa, Southern” [MeSH] OR “Africa, Western” [MeSH] OR “poverty” [MeSH]).

This strategy was adopted for use in the electronic bibliographic databases Embase and web of science.

### 2.4 Recording of studies

**Figure 2:**
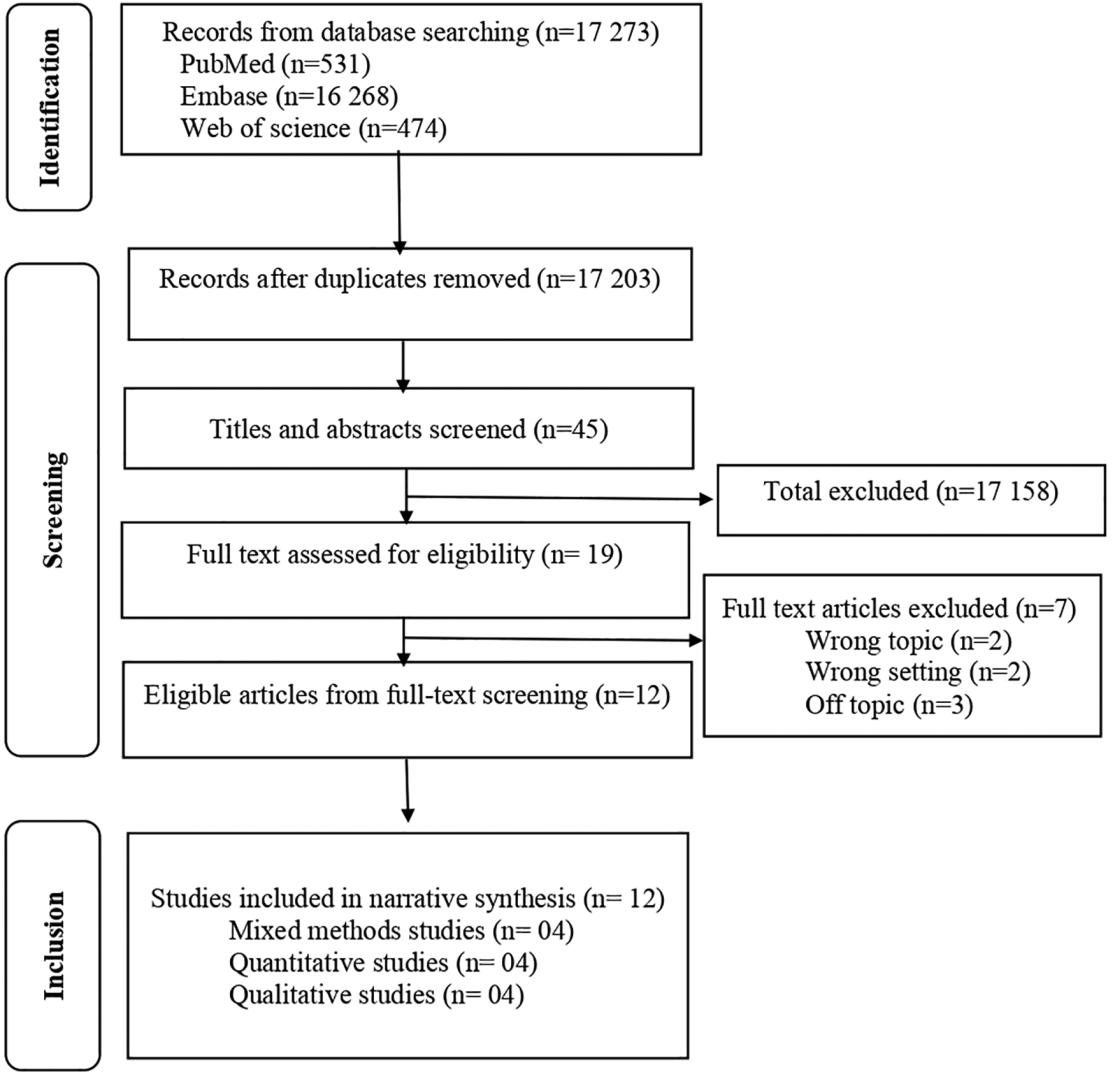
PRISMA flowchart. The selection process for the systematic review of barriers and facilitators for the sustainability of digital interventions.

The initial research yielded 17,273 studies. All identified records (*n* = 17,273) were initially reviewed by two independent researchers (SK and AB) and verified by a third researcher (PN). Retrieved studies were imported into Zotero software, ensuring the sorting of articles and the elimination of duplicates (15). And then duplicates were removed. Examining the title and abstract of the articles allowed us to exclude irrelevant articles according to the pre-established criteria. The list of relevant articles was also reviewed for additional publications. The list of records was prepared, and four researchers reviewed the full text independently (SK, AB, NB and HP). Disagreements that were linked to the inclusion were resolved by consensus involving all investigators. PRISMA flowchart was used to describe the selected studies (Figure 2).

### 2.5 Data elements

Four investigators (SK, AB, NB and HP) independently extracted data from each study that fulfilled the inclusion criteria using a standard form. In addition, for each study, the following characteristics were extracted: (1) Name of the first author; (2) Year of publication; (3) Country in which the study was conducted; (4) Setting where the study was conducted; (5) Study design; (6) Participants' characteristics; (7) Main outcomes associated with the facilitators and barriers for the sustainability of DHIs. Some of these extracted data were presented in tabular form in the results section.

### 2.6 Results and prioritization

The main outcomes are the barriers and facilitators for the sustainability of DHIs in LMICs, which can be defined as the negative and positive factors that influence the sustainability of DHIs. Barriers can be defined as negative factors that influence the sustainability of DHIs. Facilitators can be considered as the positive factors that influence positively the sustainability of DHIs. The potential influences on sustainability may derive from three major groups of factors: (1) Project design and implementation factors; (2) Factors within the organisational setting, and (3) Factors in the broader community environment (9).

### 2.7 Risk of bias in individual studies

The methodological quality of the twelve studies included in this synthesis was assessed using the Mixed Methods Appraisal Tool (MMAT) (13, 14, 16, 17). The MMAT is a critical appraisal tool designed for mixed systematic reviews, i.e., reviews that include studies using quantitative, qualitative, and mixed methods (13, 14, 16, 17). It allows the appraisal of five methodological quality categories: qualitative research, randomized controlled trials, non-randomized studies, quantitative descriptive studies, and mixed methods studies (13, 14, 16, 17). The tool is divided into two parts. First, the tool was suited for this review as it was specifically developed for quality appraisal in systematic reviews involving qualitative, quantitative and mixed methods designs. The MMAT criteria list includes indicators that explain and illustrate some criteria. For each question, the authors responded by checking “Yes”, “Don’t know”, or “No”. The methodological quality of the twelve studies included in this synthesis was assessed by four independent researchers (SK, AB, NB and HP) and verified by one researcher (PN). The authors discussed the results of the assessment for all included articles, with particular attention to questions that were checked “Don’t know”, or “No”. Qualitative and quantitative sections have four criteria each, and studies are scored by dividing the number of criteria met by four to reach at a value ranging from 25% to 100%. For mixed-method studies, we adapted the MMAT by assessing each segment separately and selecting the lowest quality rating. The MMAT is a unique tool that can be used to appraise the quality of different study designs. Also, by limiting to core criteria, the MMAT can provide a more efficient appraisal (13, 14, 16, 17). Articles were not excluded based on the MMAT score; the purpose was to examine and gain insight into rigor of existing research in this field. In terms of the methodological quality of the articles, a total of nine studies scored 5/5 (100% High quality), one study scored 4/5 (80% High quality), and two studies scored 3/5 (60% Medium quality). In general, studies were of high quality (Table 2).

### 2.8 Data synthesis

Synthesis was conducted using the information reported in the studies characteristics. Thematic content analysis was used to analyse the narrative account of the data extracted from the included studies, focused on the principle of similarity. The main findings of the studies were analysed and summarized narratively. This approach is credible and guarantees the validity of the results obtained. According to Petticrew and Roberts's approach (18), narrative synthesis was used. This technique recommends three (3) steps: (1) Organising studies in logical categories (Studies were gathered depending on the deal with factors influencing the sustainability of digital health interventions); (2) Analysis of each study (A narrative description of each study was conducted). (3) A general summary of the results of the studies was established. To organise our findings, we used Shediac-Rizkallah and Bone framework conceptualising programme sustainability (9). This framework is appropriate for this study for several relevant reasons. This framework is chosen for its multidimensional concept of sustainability because models of sustainability have been evolving to reflect challenges in the fit between intervention and context (11). On the one hand, Shediac-Rizkallah and Bone's framework the Sustainability Framework emphasises the intervention, the context of its delivery, and the broader environment within which health and healthcare systems operate (19). On the other hand, Shediac-Rizkallah and Bone's framework conceptualises sustainability using elements such as features of the project, organisational factors, and community-related factors (19). Three major factors are emphasised as potential influences on sustainability: (1) Program design and implementation factors; (2) Factors within the organisational settings; (3) Factors in the broader community environment (9). Shediac-Rizkallah and Bone's framework for conceptualising programme sustainability is presented in Figure 1. The PRISMA flowchart presenting studies examined at each stage of review is presented in Figure 2.

## 3. Results

### 3.1 Study selection

The primary research strategy yielded potentially 17,273 relevant studies. After removing duplicates and the initial screening by titles and abstracts, nineteen (19) studies were selected for full-text review. Studies were excluded if they were not LMICs studies, not limited from January 2000 to May 2022 and not focused on humans. The remaining twelve (12) studies were appraised for their methodological quality and included in the analysis. A flow chart illustrating the selection is shown in Figure 2.

### 3.2 Study characteristics

A total of twelve eligible studies were included in the systematic review. Among these studies, two were from Senegal; two were from Ghana, two were from Tanzania, one was from Jordan, one was from Malawi, one was from Ethiopia, one was from Kenya, one was from Uganda, and one was from multicentre (Ethiopia, Nigeria, and Rwanda). In addition, four used qualitative methods, four used quantitative methods and four used mixed methods. Table 1 provides a brief overview of the main characteristics of the included studies.

**Table 1:** Characteristics of included articles (*n* = 12).

No	1^er^ Author/year	Participants	Country /Setting	Study design	DHIs
01	Agoro Oscar 2018	62 men (60.2%) and 41 women (39.8%) worked in sub-county and county hospitals	Kenya	Mixed method	Electronic pharmacovigilance reporting
02	Boyce Simone Peart 2019	Health surveillance assistants using a mobile application (n = 137) and paper-based tools (n = 113), supplemented with 47 key informant interviews	Malawi/Dedza,Mzimba North, Ntcheu, and Ntchisi	Quantitative	Mobile phone application
03	Braun Rebecca 2016	25 community health workers and 148 clients	Tanzania	Mixed method	Mobile job aids
04	Diedhiou Abdoulaye 2015	Midwives, nurses, nursing assistants, health agents,	Senegal	Quantitative	mHealth
05	Downs Shauna M. 2019	Men and women	Senegal/Three rural villages	Quantitative	mHealth
06	Dusabe-Richards John N. 2016	Policy-makers and health service providers in the health facilities	Ethiopia/Three districts in the Sidama zone and one district in the Gedeo zone	Mixed method	eHealth
07	Ginsburg Amy Sarah 2016	09 health administrators, 30 health care providers, and 30 caregivers	Ghana/Six health centers and five community-based health planning and services centers	Qualitative	mHealth
08	Mangone, E. R. 2016	Mobile for Reproductive Health (m4RH) program user	Tanzania	Quantitative	mHealth
09	Mitchell-Gillespie Bria 2020	Community-Based Rehabilitation workers and Community-Based Rehabilitation managers workers	Jordan/Baqa’a Camp Community-Based Rehabilitation Center	Mixed-Method	Zoom Video Communications software
10	Motiwala F. 2021	six key players in the health technology space	Ethiopia, Nigeria, and Rwanda	Qualitative	Health technologies
11	Opoku Daniel 2019	19 Policy Makers	Ghana	Qualitative	mHealth
12	Wandera Stephen Ojiambo 2019	27 participants, including 16 key informants and 11 multi-stakeholder dialogue workshop participants	Uganda/Kampala, Jinja, and Hoima Districts of Uganda	Qualitative	health management information system

**Table 2:** Reporting the results of the MMAT.

No	Criteria from the Mixed Methods Appraisal Tool																								
	1. Qualitative					2. Quantitative randomized controlled trials					3. Quantitative non-randomized					4. Quantitative descriptive					5. Mixed methods				
	1.1	1.2	1.3	1.4	1.5	2.1	2.2	2.3	2.4	2.5	3.1	3.2	3.3	3.4	3.5	4.1	4.2	4.3	4.4	4.5	5.1	5.2	5.3	5.4	5.5
1	** **	** **	** **	** **	** **	** **	** **	** **	** **	** **	** **	** **	** **	** **	** **	** **	** **	** **	** **	** **	**1**	**1**	**1**	**1**	**1**
2	** **	** **	** **	** **	** **	** **	** **	** **	** **	** **	** **	** **	** **	** **	** **	**1**	**1**	**1**	**1**	**1**	** **	** **	** **	** **	** **
3	** **	** **	** **	** **	** **	** **	** **	** **	** **	** **	** **	** **	** **	** **	** **	** **	** **	** **	** **	** **	**1**	**1**	**1**	**1**	**1**
4	** **	** **	** **	** **	** **	** **	** **	** **	** **	** **	** **	** **	** **	** **	** **	**1**	**1**	**1**	**1**	**1**	** **	** **	** **	** **	** **
5	** **	** **	** **	** **	** **	** **	** **	** **	** **	** **	**1**	**?**	**?**	**1**	**1**	** **	** **	** **	** **	** **	** **	** **	** **	** **	** **
6	** **	** **	** **	** **	** **	** **	** **	** **	** **	** **	** **	** **	** **	** **	** **	** **	** **	** **	** **	** **	**1**	**1**	**?**	**?**	**1**
7	**1**	**1**	**1**	**1**	**1**	** **	** **	** **	** **	** **	** **	** **	** **	** **	** **	** **	** **	** **	** **	** **	** **	** **	** **	** **	** **
8	** **	** **	** **	** **	** **	** **	** **	** **	** **	** **	** **	** **	** **	** **	** **	**1**	**1**	**1**	**1**	**1**	** **	** **	** **	** **	** **
9	** **	** **	** **	** **	** **	** **	** **	** **	** **	** **	** **	** **	** **	** **	** **	** **	** **	** **	** **	** **	**1**	**1**	**1**	**?**	**1**
10	**1**	**1**	**1**	**1**	**1**	** **	** **	** **	** **	** **	** **	** **	** **	** **	** **	** **	** **	** **	** **	** **	** **	** **	** **	** **	** **
11	**1**	**1**	**1**	**1**	**1**	** **	** **	** **	** **	** **	** **	** **	** **	** **	** **	** **	** **	** **	** **	** **	** **	** **	** **	** **	** **
12	**1**	**1**	**1**	**1**	**1**	** **	** **	** **	** **	** **	** **	** **	** **	** **	** **	** **	** **	** **	** **	** **	** **	** **	** **	** **	** **

### 3.3 Barriers and facilitators for the sustainability of digital health interventions

Findings are presented in three categories according to the framework for conceptualising programme sustainability (9): (1) Project design and implementation factors; (2) Factors within the organisational settings; (3) Factors in the broader community environment.

#### 3.3.1 Project design and implementation factors level

The details of the results related to the project design and implementation factors are as follows. Among the barriers, System usability issues were most cited (20–23), especially in lower-level health facilities (23). Three studies researched and identified barriers related to internet access: Unavailable and unreliable or expensive Internet access (20, 22, 24). Two studies reported unreliable electricity in the workplace (20, 25). Two studies identified this barrier according to the limited access to computers in the workplace (20, 23). The extra time involved in using the system was noted as a barrier by two studies (20, 25). One study identified two barriers (20): First, challenges related to a hybrid system of paper and DHIs and second, the perception of no practical benefits of using the electronic system (20). Finally, another study found that insufficient understanding and infrastructure to scale up effectively and inadequate availability and accessibility of health equipment were identified as barriers (26). From the findings, facilitators were identified across studies as follows: Positive impact for improved quality healthcare delivery (21–25, 27–30); Easy use of DHIs (20, 22–24, 29, 30); Acceptability of DHIs (27, 28, 30); Availability of resources, including computers (23, 30); Feasibility of DHIs (28, 30). One study identified additional factors (30): DHIs triggered and selected according to the needs of the health system; Quality and availability of services; Accessibility of phone; Simple, the safest technologies/intervention (Apps and soft wares); Maintenance; Phone features (Screen, tailored operability); Perceived usefulness due to the functioning infrastructure (Mobile network/connectivity, transport system, electricity, basic test equipment).

At the training level, there are barriers. Capacity building issues were identified by two studies: High level of staff attrition in private facilities (23); Inadequate training in data collection and use (23), and Illiteracy rate and low level of education (30).

Many facilitators were also identified, such as empowerment of healthcare staff: Continuous training, upgrade, and education (22, 23, 30); Supportive supervision (23); and Quarterly performance review meetings (23). Three studies reported seven additional facilitators: Ready to support (30); Availability of evidence-informed (research, expert advice) (30); Availability of mHealth guidelines (30); Availability of documentation and record-keeping; Availability of awareness creation (30); Sustained knowledge gains (28); Possibility for refinement of the tool (20).

#### 3.3.2 Factors within the organisational settings

At the organisational level, integrating DHIs with existing programs/services is important. The barrier identified was Coordination challenges at the national level and changes in the structure of health management in the country, cited by one study (20). Organisational facilitators were: Priority to the DHIs at the national Healthcare system level (23); Government, institutional, sectoral, stakeholders' support (30); Availability of sustainability plan (30); Availability of staff job description (30).

#### 3.3.3 Factors in the broader community environment

At the broader community environment level, three levels were considered: (1) Socioeconomic; (2) Political consideration; (3) Community participation. The details of factors in the broader community environment are below.

##### 3.3.3.1 At the socioeconomic level

Four main barriers were identified: Limited resources (20, 21, 24, 30); Additional costs (20, 24) Abuse and corruption (30). Despite barriers, five facilitators were found across three studies: Designed to be a free and open-source platform (23); Affordability of telecommunication services (30); Availability of financial resources (30); Availability of funding mechanisms, reimbursement and incentives; and Cost-effectiveness (30).

##### 3.3.3.2 At the political consideration level

Facilitators were related to the availability of legislation and policy (Phone usage, liability, funding mechanisms and reimbursement, data security and privacy, staff job description, partners) (30) and involvement of government, institutional, sectoral, and stakeholders (30). In addition, the lack of an option for anonymous reporting in the system was identified as a barrier related to personal data protection issues (20). We also noted policy issues: inadequate policies and gaps in policy effectiveness (26).

##### 3.3.3.3 At the community participation level

From human resources, three studies identified three barriers: High level of staff attrition in private facilities and limited human resources and expertise (23); Lack of community and user integration with the technology; Unavailability of health staff and expertise (26); and Barriers related to the age (Youth ≥ ten years, adults), language, myths, fear/phobia; misconceptions (30).

There are many challenges related to usability. For example, one study identified two barriers, Low use of family planning data for planning purposes by district and health facility staff (23) and poor culture of information use (23). Another study presented two other barriers associated with usabilities, such as (20): Dislike of computer technology and Lack of a culture of pharmacovigilance reporting. Regarding behavioural barriers, one study identified one barrier related to fear of the disability-related stigma that limits the use of telehealth (22).

The findings of six studies were associated with the facilitators. The details of these facilitators are below. Government, institutional, sectoral, stakeholders' support; Penetration, and familiarity (urban) of DHIs; Locality (Urban/rural); Sociocultural acceptance; Positive attitude; Self-motivation; Age; Gender; Social class (Middle); Positive attitude interest; Dedication Willingness; Good (Provider-patient/community) relationship; Community support; Perceived ease of use; Availability of partnership and support were identified by one study (30). Two studies identified three facilitators for the sustainability of DHIs (20, 30): Triggered and selected according to the needs of communities healthcare workers; Designed and implanted with the participation of end-users; Possibility of continual feedback from end-users.

Another study highlighted two facilitators related to human resources also: The motivation and the competence of staff and Collaborative networks with the implementing partners (23, 30). In addition, to the behavioural facilitators, three facilitators were identified: Experience and confidence in using the system (22, 30); Improved satisfaction, convenience, privacy, confidentiality and trust with clients (27, 30); DHIs viewed favourably by participants (29, 30).

## 4. Discussion

The objective of this systematic review was to identify the barriers and facilitators for the sustainability of digital health intervention, especially in low and middle-income countries. Several barriers and facilitators for the sustainability of DHIs were identified during and after implementation and scale-up.

### 4.1 Main findings

DHIs play a central role in strategies to improve access, quality and efficiency of health care and services (3). Sustainability is a dynamic process, and that goals and strategies for achieving it must continuously adapt to changing environmental conditions (9). However, many DHIs have failed to become sustainable and spread across health organizations and systems (3). To our knowledge, this is the first study that has investigated the barriers and facilitators for the sustainability of DHIs in LMICs. Several barriers and facilitators for the sustainability of DHIs in LMICs were identified during and after implementation and scale-up in this review. It should be noted that there are more barriers than facilitators to the sustainability of DHIs in LMICs.

In terms of identified barriers, there are among others those associated with infrastructure, equipment, internet, electricity and the DHIs themselves. As for the facilitators, they are more focused on the strong commitment and involvement of relevant stakeholders: Government, institutional, sectoral, stakeholders' support, collaborative networks with implementing partners, improved satisfaction, convenience, privacy, confidentiality and trust with clients, experience and confidence in using the system, motivation and competence of staff. Digital health should be an integral part of health priorities and benefit people in a way that is ethical, safe, secure, reliable, equitable and sustainable. It should be developed with principles of transparency, accessibility, scalability, replicability, interoperability, privacy, security and confidentiality (1). We argue for a new approach to sustainability that instead integrates the themes of adaptive, contextually sensitive continuous quality improvement and a learning healthcare system with the challenge of intervention sustainment (11).

Regarding the factors related to the project design and implementation factors such as barriers associated with usability issues, unavailable and unreliable or expensive internet access, unreliable electricity at the workplace, limited access to computers, extra time involved in using the system, challenges associated with a hybrid system of paper and DHIs, perception of no practical benefits of using the electronic system, insufficient understanding and infrastructure to scale up effectively, and inadequate availability and accessibility of health equipment. These results corroborate those of Chirambo, Muula, & Thompson (31) for whom the telecommunication infrastructure was also found to be crucial for the mHealth decision-making program to be sustainable in Malawi. Another study noted a similar result (32). The authors revealed that the presence of the appropriate infrastructure to support the use of DHIs should be considered (32). Technological barriers compromised DHIs feasibility and DHIs stopped prematurely (33). These barriers greatly impede the effective use of DHIs or even the non-use of DHIs. Therefore, the sustainability of DHIs is not possible in these circumstances, so that, sustainability must involve: Continued learning and problem-solving, the ongoing adaptation of interventions with a primary focus on the fit between interventions and multi-level contexts, and expectations for ongoing improvement as opposed to diminishing outcomes over time (11).

At another level, the facilitators identified as follows: Positive impact for improved quality health care delivery healthcare, easy use of DHIs, acceptability of DHIs, availability of resources, including computers, the feasibility of DHIs, quality and availability of services, accessibility of phone, simple, the safest technologies/intervention (Apps and software, maintenance, phone features (Screen, tailored operability), perceived usefulness due to the functioning infrastructure (Mobile network/connectivity, transport system, electricity, basic test equipment). One study revealed a similar result (32). Indeed, the authors revealed that the presence of the appropriate infrastructure to support the use of DHIs should be considered (32). In integrating DHIs with existing programs/service levels, Clifford (34) revealed a similar result. It has been highlighted that their integration into the existing infrastructure and supporting complementary resources available is crucial in LMICs. Better conditions for sustainability of DHIs requires available, reliable and usability of appropriate material resources (Infrastructure, telecommunication, internet, computer). All these material resources create favourable conditions for the feasibility, use and acceptability of the implementation of DHIs in the perspective of their sustainability for the benefit of the populations. Reliable, secure and functional IT infrastructures and equipment are indispensable for the sustainability of DHIs in LMICs. It is therefore important to include the acquisition of reliable equipment in the sustainability plan of DIHs. An interoperable digital health ecosystem should enable the seamless and secure exchange of health data by and between users, health care providers, health systems managers, and health data services (1).

At the training level, many facilitators such as empowerment staff, continuous training, upgrade, and education, supportive supervision, quarterly performance review meetings, ready to support, availability of evidence-informed (Research, expert advice), availability of mHealth guidelines, documentation and record-keeping, awareness of the creation, sustained knowledge gains, the possibility for refinement of the tool identified in this review corroborate with one study of Labrique et al. (32). Which showed that all stakeholders must be engaged, trained and motivated to implement effectively DHIs. Because capacity-building issues increased barriers to the sustainability of DHIs. In this regard, Clifford (34) has shown that low quality of education is a real barrier to the sustainability LMICs. Dharmayat (7) found that there are many challenges to wider sustainability. In his study in Malawi, he noted that development should also focus on building local capacity by educating trainers and ensuring that training methods and guidelines are appropriately accredited based on national policies. We noted that training is one of the important keys to building strong sustainability of DHIs in LMICs. Ongoing training and refresher courses are essential to ensure that the stakeholders involved can manage DHIs effectively. DHIs should be designed and managed to include a strong training component (10). Digital health can radically change health outcomes if it is supported by sufficient investment in governance, institutional and workforce capacity to enable the changes in digital systems and data to use training that is required as health systems and services are increasingly digitized (1). Stressing the critical role played by the private sector, civil society and technical communities in information and communication technologies, it is important also to encourage strengthened and continue cooperation among stakeholders from both developed and developing countries (1).

At the organizational level, this review found that prioritising DHIs at the national Healthcare system level, government, institutional, sectoral, stakeholders' support, sustainability plan and availability of staff job description were identified as facilitators. One study found a similar result that at the global level, collaborative efforts towards a less-siloed approach to scaling and integrating digital health may provide the necessary leadership to enable innovative solutions to reach healthcare workers and patients in LMICs (32). Better quality management guided by strong leadership is needed with the close collaboration of all relevant stakeholders to ensure the sustainability of DHIs. Effective organisation is an important key to the sustainability of DHIs. DHIs should be designed and managed to (1) Demonstrate effectiveness in reaching clearly defined goals and objectives; (2) Integrate their activities fully into established administrative structures; (3) Negotiate project design with a mutually respectful process of giving and taking (10). For practice, we highlight the need for continuous assessment of the local context, not just before implementation. This enables care settings to better manage the fit between their resources, needs and interventions, including generating consistent feedback on how interventions are delivered to diverse patients and how patients do as a result (11).

Coordination challenges at the national level and changes in the structure of health management in the country are barriers. This result is close to that found in Malawi by Pérez GM, Swart W, Munyenyembe JK, Saranchuk *P*. They noted that DHIs were stopped prematurely due to organisational barriers that compromised their feasibility (33). Dharmayat found that there are many challenges to the wider implementation and sustainability, including the lack of integration with the community-based and nationwide programmes, further development, and management (7). DHIs require a significant organisational change in health systems. Continuous, participatory, inclusive and sustainable organisational change management is essential. A continuous, participatory, inclusive and sustainable organizational change management approach is more necessary than ever to accompany and guide the sustainability of DHIs. The government has an important role to play in ensuring the sustainability of DHIs at the national level. The political will of the government is a fundamental aspect for better coordination at the national level of DHIs if we consider sustainability. Ongoing stakeholder involvement throughout should lead to better sustainability. Continuously engaging stakeholders throughout the planning, implementation and adaption processes should help increase the fit between the intervention and the local context, and help address evolving issues that might interfere with sustainability (11). This applies to the relevant community stakeholders who are key actors that can effectively boost sustainability (11). Stressing the critical role played by the private sector, civil society and technical communities in information and communication technologies, it is also important to encourage strengthened and continue cooperation between and among stakeholders from both developed and developing countries (1).

According to the broader community environment level in general and in particular, at the socio-economic level, many funding issues were revealed such as limited resources, additional costs, abuse and corruption, free and open-source platform, affordability of Telecommunication services, funding mechanisms, reimbursement and incentives and cost-effectiveness. These results corroborate those of Labrique et al. (32) From whom sustainable funding is essential for supporting long-term growth, including private sector funding where appropriate. Clifford (34) in his study on LMICs found that limited funding for DHIs severely limits success. Key challenges for DHIs in lower resource environment include cost (34). Dharmayat (7)found that there are many challenges to the wider implementation and sustainability, including the absence of monetary resources (7). In this systematic review, abuse and corruption identified were not found in most studies. Fighting against corruption is the key to putting the interests of patients and public health first. Public health (35). In this regard, Madjidi & Bayubasire Ishingwa (35) noted in their study in the Democratic Republic of Congo that digital transparency systems seek to solve problems of corruption or inefficiency of services (35). Fighting corruption is key to putting the interests of patients and public health first (35). Financial resources are the lifeblood of the program. While LMICs lack sufficient financial resources to ensure the sustainability of DHIs. From planning implementation to scaling up for sustainability, financial resources must remain a given. Technical and financial support from national and international donors should be sought. Insofar as the deployment of mechanisms for mobilizing additional national and international resources will allow the financing of DHIs in LMICs. DHIs should be designed and managed to gain significant levels of funding from national sources (Budgetary and cost-recovery) during the life of the project (10). Despite the benefits of projects, factors such as a weak health system, lack of financial leadership and mentoring, and shortage of efficient health resources could prevent the continuity of funded health interventions (19). Digital health can radically change health outcomes if it is supported by sufficient investment in governance, institutional and workforce capacity to enable the changes in digital systems and data to use training, planning, and management that is required as health systems and services are increasingly digitized (1). So, the consideration of sufficient and sustainable financial resources is one of the important key to the sustainability of DHIs. To end this, the mobilisation of additional resources from development partners is essential to ensure the sustainability of DHIs in LMICs due to the scarcity of financial resources in these countries. With this essential investment in people and processes, in line with national strategies that lay out a vision for the digitalization of the health sector, digital health can improve the efficiency and cost-effectiveness of care, allowing for new business models in the delivery of services (1).

At the political level, we noted several factors such as government, institutional, sectoral, stakeholders' support, legislation and policy (Phone usage, liability, funding mechanisms and reimbursement, anonymous reporting in the system, inadequate policies and gaps in policy effectiveness, data security and privacy, staff job description, partners), involvement of government, institutional, sectoral, stakeholders. These results are close to that found by Labrique et al. (32). They noted that the policy environment in which DHIs are intended to function, where alignment with broader healthcare policy, is essential (32). In addition, stakeholders who are engaged in sustainable mHealth programmes in resource-poor settings can be used to develop an evidence-based policy for the utilization of technology for healthcare delivery across developing countries (32). Chirambo, Muula, & Thompson (31) in Malawi revealed that the sustainability of mHealth tools for children under 5 care can depend on a robust level of political commitment from the government working in collaboration with NGOs involved in these technologies. Strong political commitment is more than ever necessary for a better accompaniment and good guidance for the sustainability of DHIs in LMICs. Close collaboration between the government and all relevant stakeholders is one of the conditions for the sustainability of DHIs. It is more than ever essential that there be a strong commitment from the government in collaboration with development partners to accompany the establishment, scaling up and sustainability of DHIs in LMICs. Creating an enabling policy environment for the development of DHIs is crucial.

Community participation is facing many challenges related to high levels of staff attrition in private facilities, limited human resources and expertise, lack of community and user integration with the technology, unavailability of health staff and expertise, and barriers related to age, language, myths, fear/phobia, and misconceptions. These challenges make it difficult for sustainability because community mobilisation was recognized by many of the reviewed studies as a crucial facilitator for intervention sustainability, both early on and after intervention implementation (8). Community participation is fundamental to the effective sustainability of DHIs. But, many challenges related to usability were shown. For example, low use of family planning data for planning purposes by district and health facility staff, the poor culture of information use, dislike of computer technology, lack of a culture of pharmacovigilance reporting and fear of the disability-related stigma that limits the use of telehealth. These results are close to the result of one study that found a reason such as a difficulty to use technology. These results are close to the result of one study that found a reason such as a difficulty to use technology (36). All the challenges associated with the use of DHIs are human and cognitive. Human-technology interactions are not without their challenges. The acceptability and usability of DHIs, which are fundamental to ensuring sustainability, depend largely on the end-users. Communities must be well-equipped.

Facilitators associated with the government, institutional, sectoral, stakeholders' support, penetration, and familiarity (Urban) of DHIs, sociocultural acceptance, positive attitude, self-motivation; age, gender, social class (Middle), positive attitude interest, dedication, willingness, Good (Provider-patient/community) relationship, community support, perceived ease of use, availability of partnership and support identified in this review corroborate with findings from Akeju et al. (5). They showed that four project outcomes that were achieved at end-line evaluation were sustained 12 months after project close down, namely: Staff motivation and satisfaction; increased staff confidence to perform healthcare roles; improved standard of healthcare delivery; and increased adoption of DHIs beyond the health sector.

Facilitators for the sustainability of DHIs such as triggered and selected according to the needs of communities' healthcare workers, designed and implanted with the participation of end-users, and the possibility of continual feedback from end-users are similar to results found in the study of Labrique et al. (32). Their findings revealed that the intrinsic characteristics of DHIs must offer tangible benefits to address an unmet need, with end-user input from the outset. We view sustainability as akin to the challenge of fitting a puzzle piece within an evolving large tableau. Without sensitivity to the characteristics of the intervention, practice setting, and the larger system, there is little expectation that the intervention will fit well within the setting, and as the context changes, sustainment will be harder and harder to achieve (11).

Regarding motivation and competence of staff, collaborative networks with implementing partners, experience and confidence in using the system, improved satisfaction, convenience, privacy, confidentiality and trust with clients, and viewed favourably by actors were corroborated with the results of Labrique et al. (32). They agreed that all stakeholders must be engaged, where collaborated and motivated to implement effectively DHIs. Committed participation of stakeholders is required in LMICs (32). Successful sustainability inevitably requires the effective participation of all relevant stakeholders at all stages of implementation, scaling up and sustainability of DHIs. All relevant stakeholders must be involved in the implementation, scaling up and sustainability of DHIs. Adaptation is expected and even encouraged. Assessment of care settings and outcomes is ongoing and incorporated within the practice, and staffing and policy changes are incorporated in sustainability planning (11). Health-related projects are highly beneficial in the restoration, and preservation of community health, and could be sustained by factors such as community ownership working within existing resources, and training (19). The global digital strategy emphasizes that health data are to be classified as sensitive personal data, or personally identifiable information, that requires a high safety and security standard (1). Therefore, it stresses the need for a strong legal and regulatory base to protect privacy, confidentiality, integrity and availability of data and the processing of personal health data, and to deal with cybersecurity, trust building, accountability and governance, ethics, equity, capacity building and literacy, ensuring that good quality data are collected and subsequently shared to support planning, commissioning and transformation of services (1). It is important to maintain transparency and effectively communicate data security strategies (1). In conclusion, digital health will be valued and adopted if it: is accessible and supports equitable and universal access to quality health services; enhances the efficiency and sustainability of health systems in delivering quality, affordable and equitable care, and strengthens, and scales up health promotion, disease prevention, diagnosis, management, rehabilitation, and palliative care including before, during and after an epidemic or pandemic, in a system that respects the privacy and security of patient health information (1). The vision of the global strategy is to improve health for everyone, everywhere by accelerating the development and adoption of appropriate, accessible, affordable, scalable, and sustainable person-centric digital health solutions to prevent, detect and respond to epidemics and pandemics, developing infrastructure and applications that enable countries to use health data to promote health and well-being, and to achieve the health-related Sustainable Development Goals (1). To face the different challenges, it is necessary to act efficiently. Recognize the urgent need to address the major impediments faced by least-developed countries implementing digital health technologies (1). There is a pressing need to invest in efforts to overcome the major impediments that developing countries face in engaging with and accessing new digital health technologies, such as an appropriate enabling environment, sufficient resources, infrastructure to support the digital transformation, education, human capacity, financial investment and internet connectivity, as well as issues related to legacy infrastructure, technology ownership, privacy, security, and adapting and implementing global standards and technology flows (1).

WHO recommends four strategic objectives intended to provide guidance and coordination on global digital health transformation and to strengthen synergies between initiatives and stakeholders to improve health outcomes and mitigate associated risks at all levels: (1) Promote global collaboration and advance the transfer of knowledge on digital health; (2) Advance the implementation of national digital health strategies; (3) Strengthen governance for digital health at global, regional and national levels; (4) Advocate people-centred health systems that are enabled by digital health. Monitoring and evaluation of DHIs must be at the heart of the implementation, scaling up and sustainability of DHIs to achieve the expected results at all levels. It is important to dynamically monitor the maturity level of digital health in countries and institutions and to assess the implementation of digital health strategies through standard agreed-upon metrics (1). These measures should include both the status and performance of digital health interventions and include established monitoring and evaluation models to facilitate monitoring of the contribution of digital health to health system processes, health workforce processes and individual health needs (1). Consideration should be given to align the digital health performance monitoring indicators with a national action plan for linking the global strategy on digital health and action plan with policy options and actions, outputs, outcomes and impacts (1).

Especially according to health and human rights on DHIs and the right to health, UNDP guidance on rights-based use of DHIs highlighted that Digital inclusion is essential because it is an approach to close divides in access to and use of digital technologies (37). A practice which ensures that all individuals and communities, including the most disadvantaged, are aware of, have access to, and use/are able to use information and communication technologies as well as needed, relevant, and safe digital content and services (37). Global norms and standards are also essential: Convene partners for dialogues to develop and bridge country-level best practices to the development and implementation of global norms and standards, including for data privacy and protection, ethics, and human rights (37). Finally, Inclusive/People-centred digital transformation is very important: An approach that puts people at the centre of digital transformation efforts to ensure a more open, transparent, and accessible process. For example, UNDP, advocates that inclusive digital transformation: (1) Addresses the needs of the poorest as well as the most vulnerable and marginalized groups, including women and people with disabilities; (2) Mitigates the tendency of digital transformation to exacerbate existing inequalities; (3) Empowers underrepresented groups to take part in meaningful ways; (4) Protects people from the adverse effects of digital technologies; (5) Encourages the use and development of digital technology that is open, responsible, and rights-based (37).

### 4.2 Importance for research and practice

The review has provided an understanding of challenge about the sustainability of DHIs in LMICs. Simultaneously, the review has highlighted the barriers (Poor technological literacy, insufficient network coverage, high cost of service and expectations not favouring the use of DHIs and facilitators, including program design and implementation factors, factors within the organisational settings and factors in the broader community environment. Additionally, larger-scale and more rigorous studies are needed to assess sustainability of DHIs. Despite these research needs, DHIs have significant potential to alter the landscape for well-being of people in LMICs and is worthy of attention and support. This opens a window to examine the issue from a broader perspective and explore the most important sustainability of DHIs Challenges in LMICs. Finally, future research should explore new areas of sustainability of DHIs.

### 4.3 Limitations of the study

This systematic review has its strengths and weaknesses. To ensure a comprehensive search strategy, we used a literature search strategy adding specific termes for the four components we were interested in studying (Digital Health Intervention, sustainability, barriers and facilitators) and used explicit inclusion and exclusion criteria. We identified four limitations in this review. First, despite applying a comprehensive research strategy, it is possible that not all relevant studies were retrieved and included in this review. Secondly, although we included only papers published in peer-reviewed journals to improve the review's quality, this may have resulted in the omission of outside reports from grey literature or papers published in technology journals. Important sources of information on barriers and facilitators for the sustainability of DHIs may exist as grey literature and are inaccessible because they have not been published in the websites consulted or encoded in the databases used. Not all databases were searched, and no further contact with the authors of included papers was made. Third, as only twelve primary research were included, it is impossible to draw adequate conclusions based on the limited amount and quality of evidence available. Fourth, another limitation is that we only included articles published in English and French.

### 4.4 Recommendations

The results of this systematic review point out to some implications. Consideration of potential barriers and facilitators should inform DHIs projects, from their planning phase through to their scaling up and beyond. It would be appropriate at the health system level to consolidate facilitators and to manage efficiently barriers with all stakeholders. Therefore, it becomes needful that identified facilitators of sustainability are promoted, while the impediments are immediately addressed through some strategies (19). This will help to improve the design, implementation, scale-up, and sustainability of DHIs. In light of the above implications, it is important to develop strategies to improve the latter's effectiveness and increased attention to the sustainability of DHIs.

For the successful sustainability of DHIs in LMICs, the ministry in charge of health in close collaboration with the ministry in charge of technology, decision-makers, partners and relevant stakeholders should promote: (1) Proper governance to build a strategy for sustainability of DHIs. So that, rethinking governance to better ensure the sustainability of DHIs is important; (2) Strong commitment of decision makers to frame decision-making for the implementation, scale-up and sustainability of DHIs; (3) Ongoing and appropriate funding for sustainability of DHIs; (4) Adequate technologies and technological environments to have good technical conditions of work and use; (5) Technical profile of the DHIs driven by simplicity, interoperability and adaptability to facilitate user acceptance and use; (6) Participatory and collaborative of all stakeholders for improved acceptability and use of DHIs; (7) Pluralistic management approach of all stakeholders is necessary to achieve good governance, high level of involvement of relevant stakeholders and high level of acceptability and use by end users; (8) Organisational support for the proper functioning of DHIs is important because an appropriate organisation always facilitates the feasibility, implementation, scaling-up and sustainability of DHIs; (9) Sustainability plan institutionalisation of DHIs to declare all activities aimed at the sustainability of DHIs. This sustainability plan should be well-developed, monitored and periodically evaluated in collaboration with all relevant stakeholders. Thus, all activity managers will work towards the timely completion of all activities for the full success of the technological and digital health projects; (10) Continuous training, retraining and continuous development of all stakeholders to support the implementation, scaling up and sustainability of DHIs; (11) Systematic continuous monitoring during and after implementation and scale-up is to be seriously considered for capacity building of key actors involved in the deployment of DHIs; (12) Systematic evaluation of each DHIs before, during and after implementation and scale-up of DHIs to ensure effective monitoring of DHIs. This is essential to highlight the barriers to be managed in time and the facilitators to be consolidated; (13) Active community participation is fundamental to involve all stakeholders in the sustainability of DHIs; (14) Use of available evidence of research about barriers and facilitators to better designing, implementation, scale-up and sustainability of DHIs. Research is a powerful lever for the development of effective and sustainable digital health interventions. The sustainability of digital health interventions inevitably requires research. Evidence on the issues and challenges of sustainability of digital health interventions is widely documented through scientific research. This allows awareness of these issues and challenges by the actors involved in the development of digital health interventions in order to better manage the issue of sustainability of digital health interventions; (15) Use effective tools for effective evaluation of DHIs. Availability of reliable DHIs assessment tools makes it possible to conduct regular and effective assessments; (16) Development of more tools for effective evaluation of DHIs taking into account factors that influence sustainability of DHIs. As the persistence of DHIs is a dynamic process, it is necessary to have tools that adapt to this evolutionary dynamic according to the different parameters to be considered; (17) There is a need for more scientific studies in this area as digital health interventions are complex and variable. The advancement of knowledge in digital health is very useful.

For full acceptability, usability and effective participation of relevant stakeholders involved in the process of implementation, scaling up and sustainability of DHIs, it remains very important to (1) Design and develop DHIs in line with the real needs of the population. In this case, the DHIs will be of real interest to the whole population. In this regard, it will work tirelessly for the success of DHIs insofar as it provides them with appropriate solutions to their health problems; (2) Involve relevant stakeholders in the process of implementing, scaling up and sustaining DHIs. The involvement of all relevant stakeholders is a prerequisite for the successful implementation of DHIs; (3) Promote awareness for effective use through mass media. Continuous awareness raising of all relevant stakeholders is very important in all social networks to reach the maximum number of key actors; (4) Promote training, and effective retraining of all relevant stakeholders involved in the process of implementation, scaling up and the sustainability of DHIs. Training remains essential for all relevant stakeholders. They aim at strengthening their capacities. This enables these key actors to be actively and effectively involved; (5) Promote feedback from end-users, health personnel, and technology teams on the interaction between actors and technology. Feedback from users allows us to be aware of their real concerns to better manage them effectively with their full collaboration; (6) Ensure that relevant stakeholders see their real interests and especially the usefulness of DHIs.

## 5. Conclusion

This systematic review allowed us to identify barriers and facilitators to the sustainability of DHIs in LMICs. The results showed that sustainability is multifactorial. Factors used can be grouped into three categories according to the framework for conceptualizing programme sustainability: Project design and implementation, organizational, and broader community environmental factors. All stakeholders play an important role in the sustainability process. The review provides insights for the research community and all stakeholders in making decision regarding sustainability of DHIs in LMICs. The findings from this systematic review provide a common ground, making it possible to understanding better the challenges and opportunities related to sustainability of DHIs. These results help all stakeholders to improve the sustainability of DHIs. In addition, these results will make it possible to better monitor and evaluation of DHIs during implementation and scale-up with stakeholders' participation and rethinking of the conception and development of DHIs.

## Data Availability

'The original contributions presented in the study are included in the article/Supplementary Materials, further inquiries can be directed to the corresponding author/s.
