# Supplementary material for: Barriers and facilitators for the sustainability of digital health interventions in low and middle-income countries: A systematic review
Source: Front Digit Health. 2022 Nov 28;4:1014375. doi: 10.3389/fdgth.2022.1014375 (PMC9742266; doi:10.3389/fdgth.2022.1014375)
Supplement: Supplementary file 1 [file Table3.docx]

**Table 3: Barriers and facilitators identified in included articles (n=12)**

| **Dimensions** | **Barriers** | | **Facilitators** |
| --- | --- | --- | --- |
| **Project design and implementation factors** | | | |
| **Project effectiveness** | **Agoro, O. O., Kibira, S. W., Freeman, J. V., & Fraser, H. S. F. (2018)** | | |
|  | No practical benefits of using the electronic system- Unavailable, unreliable or expensive Internet access **-**Unreliable electricity at the workplace-Limited access to computers at the workplace - Extra time involved in using the system - System usability issues | | Usability, Ease of use of DHI |
|  | **Motiwala, F., & Ezezika, O. (2021)** | | |
|  | Insufficient understanding and infrastructure to scale up effectively **-**Inadequate availability and accessibility of health equipment | | None |
|  | **Ginsburg, A. S., Tawiah Agyemang, C., Ambler, G., Delarosa, J., Brunette, W., Levari, S., … Anderson, R. (2016)** | | |
|  | Unreliable electricity at the workplace **-** Extra time involved in using the system | | Positive impact for improved quality health care delivery healthcare |
|  | **Wandera, S. O., Kwagala, B., Nankinga, O., Ndugga, P., Kabagenyi, A., Adamou, B., & Kachero, B. (2019)** | | |
|  | Limited access to computers at the workplace- System usability issues especially in lower level health facilities | | Positive impact for improved quality health care delivery healthcare - Usability, Ease of use of DHI Availability of resources, including computers |
|  | **Mitchell-Gillespie, B., Hashim, H., Griffin, M., & AlHeresh, R. (2020)** | | |
|  | Unavailable, unreliable or expensive Internet access-System usability issues | | Positive impact for improved quality health care delivery healthcare **-**Usability, Ease of use of DHI |
|  | **Dusabe-Richards, J. N., Tesfaye, H. T., Mekonnen, J., Kea, A., Theobald, S., & Datiko, D. G. (2016)** | | |
|  | Unavailable, unreliable or expensive Internet access- Limited power sources, especially in rural areas - System usability issues | | Positive impact for improved quality health care delivery healthcare **-**  Usability, Ease of use of DHI **-**  Availability of resources, including computers **(Wandera) (Opuku)**  Acceptability of DHIs **(Braun) (Diedhiou)** **(Opuku)**  Feasibility of DHIs (**Diedhiou)** **(Opuku)** |
|  | **Diedhiou, A., Gilroy, K. E., Cox, C. M., Duncan, L., Koumtingue, D., Pacqué-Margolis, S., … Bailey, R. (2015)** | | |
|  |  | | Acceptability of DHIs - Feasibility of DHIs |
|  | **Downs, S. M., Sackey, J., Kalaj, J., Smith, S., & Fanzo, J. (2019)** | | |
|  |  | | Positive impact for improved quality health care delivery healthcare **-**Usability, Ease of use of DHI |
|  | **Braun, R., Lasway, C., Agarwal, S., L’Engle, K., Layer, E., Silas, L., … Kudrati, M. (2016)** | | |
|  |  | | Positive impact for improved quality health care delivery healthcare -Acceptability- Improved privacy, confidentiality and trust with clients  Both CHWs and their clients reported that the mobile job aid was a highly acceptable FP support tool. CHWs perceived benefits to  service quality, including timelier and more convenient care; better quality of information; increased method choice; and improved privacy,  confidentiality and trust with clients |
|  | Boyce, S.P., Nyangara, F., & Kamunyori, J. (2019) | | |
|  | System usability issues | | Positive impact for improved quality health care delivery healthcare |
|  | Opoku, D., Busse, R., & Quentin, W. (2019) | | |
|  | - | | Positive impact for improved quality health care delivery healthcare **-** Usability, Ease of use of DHI  Availability of resources, including computers **-** Acceptability of DHIs**-**Feasibility of DHIs - Quality, availability and affordability of services - accessibility of phone - Simple, safest and easy technologies/ intervention (apps and softwares) -type of (available) technologies - maintenance - phone features (screen, tailored operability) - Triggered and selected according to the needs of the health system: Positive impact for improved quality health care delivery healthcare- Perceived usefulness : Functioning infrastructure (mobile network/connectivity, transport system, electricity, basic test equipment) |
| **Training** | Wandera, S. O., Kwagala, B., Nankinga, O., Ndugga, P., Kabagenyi, A., Adamou, B., & Kachero, B. (2019) | | |
|  | Capacity building issues: high levels of staff attrition in private facilities **-** inadequate training in data collection and use | | Training staff **-** supportive supervision and quarterly performance review meetings |
|  | Opoku, D., Busse, R., & Quentin, W. (2019) | | |
|  | Illiteracy and low level of education | | Ready to support - Continuous training, upgrade, and education -Evidence-informed (research, expert advice) - Awareness creation  - Availability of mHealth guidelines |
|  | Diedhiou, A., Gilroy, K. E., Cox, C. M., Duncan, L., Koumtingue, D., Pacqué-Margolis, S., … Bailey, R. (2015) | | |
|  | - | | Sustained knowledge gains |
|  | Agoro, O. O., Kibira, S. W., Freeman, J. V., & Fraser, H. S. F. (2018) | | |
|  | Lack of a culture of pharmacovigilance reporting -Dislike of computer technology - Lack of support/incentives from management to use the system for reporting -Lack of awareness of existence of the electronic reporting system -  - | | Possibility for refinement of the tool  Possibility of continual feedback from end users |
|  | Mitchell-Gillespie, B., Hashim, H., Griffin, M., & AlHeresh, R. (2020) | | |
|  | - | | Initial and ongoing user training |
| **Factors within the organizational settings** | | | |
| **Integration with existing programs/services** | Agoro, O. O., Kibira, S. W., Freeman, J. V., & Fraser, H. S. F. (2018) | | |
|  | Coordination challenges at the national pharmacovigilance center and changes in the structure of health management in the country also had an impact on the success of the electronic reporting system | | - |
|  | Wandera, S. O., Kwagala, B., Nankinga, O., Ndugga, P., Kabagenyi, A., Adamou, B., & Kachero, B. (2019) | | |
|  |  | | Organizational facilitators: Prioritizing family planning data |
|  | Opoku, D., Busse, R., & Quentin, W. (2019) | | |
|  |  | | Government, institutional, sectoral, stakeholders’ support |
| **Factors in the broader community environment** | | | |
| **Socio-economic** | Dusabe-Richards, J. N., Tesfaye, H. T., Mekonnen, J., Kea, A., Theobald, S., & Datiko, D. G. (2016) | | |
|  | Additional costs recharge fees | |  |
|  | Agoro, O. O., Kibira, S. W., Freeman, J. V., & Fraser, H. S. F. (2018) | | |
|  | Extra cost of electronic reporting (Internet data costs) | |  |
|  | Boyce, S.P., Nyangara, F., & Kamunyori, J. (2019 | | |
|  | Limited resources | |  |
|  | Ginsburg, A. S., Tawiah Agyemang, C., Ambler, G., Delarosa, J., Brunette, W., Levari, S., … Anderson, R. (2016) | | |
|  |  | | Designed to be a free and open-source platform |
|  | Motiwala, F., & Ezezika, O. (2021) | | |
|  | Lack of financial resources | | - |
|  | Mangone, E. R., Agarwal, S., L’Engle, K., Lasway, C., Zan, T., van Beijma, H., … Karam, R. (2016) | | |
|  |  | | Incorporation of successful and socially desirable business models that can lead to profit generation |
|  | Opoku, D., Busse, R., & Quentin, W. (2019) | | |
|  | Abuse/corruption | | Affordability of (telecommunication) services - Avaibility of financial resources, funding mechanisms, reimbursement and incentives-  Cost-Effectiveness |
| **Political considerations** | Motiwala, F., & Ezezika, O. (2021) | | |
|  |  | | Policies issues: inadequate policies and gaps in policy effectiveness |
|  | Opoku, D., Busse, R., & Quentin, W. (2019) | | |
|  | Personal data protection issues: Lack of an option for anonymous reporting in the system |  | Perceived ease of use due to legislation and policy - Legislation and policy (phone usage, liability, funding mechanisms and reimbursement, data security and privacy, staff job description, partners) - Government, institutional, sectoral, stakeholders’ support |
| **Community participation** | Agoro, O. O., Kibira, S. W., Freeman, J. V., & Fraser, H. S. F. (2018) | | |
|  |  | | Designed and implanted with the participation of end users **-**Triggered and selected according to the needs of CHWs - |
|  | Wandera, S. O., Kwagala, B., Nankinga, O., Ndugga, P., Kabagenyi, A., Adamou, B., & Kachero, B. (2019) | | |
|  | Limited human resources and expertise **-** High levels of staff attrition in private facilities **-** Low use of family planning data for planning purposes by district and health facility staff-Poor culture of information use | | Motivation and competence of Staff**-**Collaborative networks with implementing partners |
|  | Motiwala, F., & Ezezika, O. (2021) | | |
|  | Lack of community and user integration with the technology **-**Unavailability of health personnel and expertise | | - |
|  | Mitchell-Gillespie, B., Hashim, H., Griffin, M., & AlHeresh, R. (2020) | | |
|  | Fear of disability-related stigma may limit the use of telehealth in this setting | | Experience and confidence in using the system |
|  | Braun, R., Lasway, C., Agarwal, S., L’Engle, K., Layer, E., Silas, L., … Kudrati, M. (2016) | | |
|  |  | | Improved privacy, confidentiality and trust with clients |
|  | Downs, S. M., Sackey, J., Kalaj, J., Smith, S., & Fanzo, J. (2019) | | |
|  | - | | DHIs viewed favourably by participants |
|  | Opoku, D., Busse, R., & Quentin, W. (2019) | | |
|  | Language- myths, fear/phobia, misconceptions  - age (youth ≥10 years, adults)- | | Government, institutional, sectoral, stakeholders’ support- Informed, convinced, trust, and confidence (satisfaction) - Locality (urban/rural)- Socioculture -Acceptance - Positive attitude - Self-motivation –Age-Gender -Social class (middle) - Positive attitude interest- Dedication –Willingness- Good (provider-patient/community) relationship- Confidence- Basic, Simple-Personalization- Perceived ease of use: penetration, and familiarity (urban) -Partnership and support- Convenience - Confidentiality and privacy - Community support -Data security and privacy |
